# An excessive increase in glutamate contributes to glucose-toxicity in β-cells via activation of pancreatic NMDA receptors in rodent diabetes

**DOI:** 10.1038/srep44120

**Published:** 2017-03-17

**Authors:** Xiao-Ting Huang, Chen Li, Xiang-Ping Peng, Jia Guo, Shao-Jie Yue, Wei Liu, Fei-Yan Zhao, Jian-Zhong Han, Yan-Hong Huang, Y -L Yang-Li, Qing-Mei Cheng, Zhi-Guang Zhou, Chen Chen, Dan-Dan Feng, Zi-Qiang Luo

**Affiliations:** 1 Department of Physiology, Xiangya School of Medicine, Central South University, Changsha, Hunan, China; 2 Department of Physiology, Changzhi medical college, Changzhi, Shanxi, China; 3 Xiangya Nursing School, Central South University, Changsha, Hunan, China; 4 Department of Pediatrics, Xiangya Hospital, Central South University, Changsha, Hunan, China; 5 Department of Metabolism and Endocrinology, the Second Xiangya Hospital, Central South University, Changsha, Hunan, China; 6 SBMS, The University of Queensland, Brisbane, Australia

## Abstract

In the nervous system, excessive activation of NMDA receptors causes neuronal injury. Although activation of NMDARs has been proposed to contribute to the progress of diabetes, little is known about the effect of excessive long-term activation of NMDARs on β-cells, especially under the challenge of hyperglycemia. Here we thoroughly investigated whether endogenous glutamate aggravated β-cell dysfunction under chronic exposure to high-glucose *via* activation of NMDARs. The glutamate level was increased in plasma of diabetic mice or patients and in the supernatant of β-cell lines after treatment with high-glucose for 72 h. Decomposing the released glutamate improved GSIS of β-cells under chronic high-glucose exposure. Long-term treatment of β-cells with NMDA inhibited cell viability and decreased GSIS. These effects were eliminated by *GluN1* knockout. The NMDAR antagonist MK-801 or *GluN1* knockout prevented high-glucose-induced dysfunction in β-cells. MK-801 also decreased the expression of pro-inflammatory cytokines, and inhibited I-κB degradation, ROS generation and NLRP3 inflammasome expression in β-cells exposed to high-glucose. Furthermore, another NMDAR antagonist, Memantine, improved β-cells function in diabetic mice. Taken together, these findings indicate that an increase of glutamate may contribute to the development of diabetes through excessive activation of NMDARs in β-cells, accelerating β-cells dysfunction and apoptosis induced by hyperglycemia.

Diabetes affects 8.3% of adults worldwide and its morbidity is increasing. Diabetes has become one of the most common non-communicable diseases in the current era^1^. In diabetes, islet dysfunction is associated with the loss of β-cell mass and a decrease in insulin secretion, occurring not at the onset but rather as a consequence of diabetes and hyperglycemia^2^. Loss of function and/or mass β-cells is partially due to glucotoxicity, which is defined as long-term exposure to a hyperglycemic environment, leading to the loss of β-cells function and reduced β-cells differentiation^3^. However, the exact mechanisms underlying the dysfunction of β-cells induced by hyperglycemia remain unclear.

Imbalance of metabolic regulatory systems is the basis for many metabolic disorders, including diabetes^4^. Although the evidence indicates that diabetes affects the metabolism of amino acids^5^,^6^, the converse effect of amino acid metabolism on diabetes is unclear. Glutamate is an important excitatory neurotransmitter^7^. Excessive activation of glutamate receptors evokes excitatory neurotoxicity in neurons^8^. Glutamate receptors, which include more than twenty subtypes, have been classified into two major categories: the ionotropic glutamate receptors (that function as ion channels) and the metabotropic glutamate receptors^8^. Glutamate neurotoxicity is primarily mediated by N-methyl-D-aspartate (NMDA) receptors, which belong to the family of ionotropic glutamate receptors^9^. Recently, NMDARs have been found in peripheral non-neuronal tissues and cells, including the kidney, lung, urogenital tract and pancreatic β-cells^10^,^11^,^12^.

As pancreatic islet β-cells share many cell biology features with neurons^13^, NMDARs may play an important role in the viability and function of β-cells. However, the literature remains controversial. NMDA elicits a rise in [Ca^2+^]_i_ in single β-cells *in vitro*^14^, and causes transient insulin secretion^15^. The NMDA induced rises in [Ca^2+^]_i_ are suppressed by the NMDAR antagonist, MK-801^10^,^14^. A recent study has shown that treatment with the NMDAR antagonist dextromethorphan (DXM) improves islet insulin secretion and blood glucose control in type 2 diabetes mellitus^16^. This suggests that NMDAR may be a potential target for treatment of diabetes. However, the effects of long-term NMDAR activation on the function of β-cells under chronic hyperglycemia in diabetes are still unclear. We have reported that endogenous glutamate was selectively released from the lungs in acute lung injury induced by bleomycin or hyperoxia. Blocking NMDARs attenuates lung injury induced by bleomycin or hyperoxia through inhibition of inflammation and oxidative stress^17^,^18^. Because inflammation and oxidative stress also play important roles in the development and progression of diabetes, we hypothesize that long-term activation of NMDARs leads to the dysfunction of β-cells *via* aggravation of the inflammation and oxidative stress induced by hyperglycemia in diabetes.

In this study, we found that plasma glutamate was increased in diabetic patients and mice. Long-term treatment with exogenous NMDA caused dysfunction in β-cell lines, and blockade of NMDAR alleviated the damage to β-cells induced by glucotoxicity *in vitro*. Furthermore, the NMDAR antagonist Memantine decreased blood glucose concentrations in diabetic mice by improving the function of β-cells, and this effect was due to a reduction in the NOD-like receptor family member pyrin domain-containing protein 3 (NLRP3) inflammasome and NF-κB levels in β-cells.

## Methods

### Human Sample Collection

Patients (n = 32) were recruited from the community of Wang Yue Hu, Changsha, China. Inclusion criteria included living with type 2 diabetes for at least 6 years. Exclusion criteria included cancer, stroke, thrombosis or myocardial infarction within the previous 3 months. Most of the patients in the study received the drug treatment: 12 patients received Metformin, 7 received Rosiglitazone and 7 received insulin injection. Another twenty medically cleared cases were recruited to the normal group. Weight and height were measured with a standardized method by the same physician. Body mass index (BMI) was calculated as the body weight (kg) divided by the square of the height (m). No significant difference was noted between the groups in terms of gender, ethnic origin, age or BMI. Blood samples were collected after an overnight fast of 8–10 h. Glycated hemoglobin (HbA1c) was determined by high-performance liquid chromatography (Tosoh, Japan). The characteristics of normal individuals and patients in the study were shown in Table 1. Plasma glutamate levels were also measured by high-performance liquid chromatography.

This study was approved by the institutional review board of Central South University Xiangya Hospital. All analyses were performed in accordance with relevant guidelines and regulations. Written informed consent was obtained from each participant.

### Ethics statement

The Ethics Committee of the Center for Scientific Research with Animal Models at Central South University (Changsha, China) approved the experiments, which were performed in accordance with the guidelines of the National Institutes of Health. Mice were anesthetized with chloral hydrate (400 mg/kg, intraperitoneally) and necessary efforts were taken to minimize suffering before performing operations.

### Animal Model

Male Balb/c mice (8-week old) were given a standard diet and water. After a one-week adaptation period, mice were fed with a high-fat (60% calories) diet for 2 weeks. Diabetes was then induced by a single intraperitoneal injection of streptozotocin (STZ, Sigma, USA) in citrate-phosphate buffer (0.1 M, pH 4.2) at a dose of 100 mg/kg after 4 h of fasting^19^,^20^. And control mice were received a single intraperitoneal injection of citrate-phosphate buffer (0.1 M, pH 4.2) after 4 h of fasting. Hyperglycemia was assessed by measuring the fasting serum glucose (FSG) levels of the mice 72 h after STZ administration. Mice injected of STZ with FSG levels higher than 13.89 mmol/L were selected for subsequent experiments.

After diabetes was induced, mice were divided into 4 groups: control group (normal mice received intraperitoneal injection with saline for 3 weeks), Memantine group (normal mice received intraperitoneal injection with Memantine for 3 weeks, 10 mg/kg/day), diabetes group (diabetic mice received intraperitoneal injection with saline for 3 weeks) and Memantine + diabetes group (diabetic mice received intraperitoneal injection with Memantine for 3 weeks, 10 mg/kg/day). The four groups of mice were all fed a high-fat diet for 9 weeks.

### Islet Isolation

To isolate primary islets from male SD rat (8-week old, 200–250 g), the bile duct near the ampulla of vater was ligated, and the common bile duct was cannulated and injected with 10 mL of Hanks’ buffer containing collagenase V (1 mg/mL, Sigma, USA). The pancreas was dissected from the surrounding tissues, removed, and incubated in a stationary bath at 37 °C for 15 min. The digested tissue was washed with Hanks’ buffer without collagenase, and then the islets were purified by a density gradient (Histopaque 1077 and Hanks’ buffer; Sigma, USA) by centrifugation at 3,000 g for 20 min^2^. Islets were collected and incubated at 37 °C.

### RNA isolation and real-time PCR

Total RNA from pancreas or β-cells was extracted using TRIzol reagent (Invitrogen, USA) and reverse transcribed by RevertAidTM Reverse Transcriptase (Thermo-Scientific, USA). Gene expression levels were assayed by real-time PCR using a SYBR Green PCR Master Mix (Biorad, USA) on a Bio-Rad real-time PCR system (CFX96 Touch™, Bio-Rad, USA). The levels of mRNA were normalized to β-actin. The primers for the targeted genes are detailed in Supplementary Table 1 (Supplementary information).

### Cell culture of MIN6/RINm5f cells

MIN6 cells or RINm5f cells were cultured in DMEM (HyClone, USA) or RPMI 1640 (HyClone, USA) and supplemented with 10% fetal bovine serum (Gibco, USA). They were passaged and harvested using trypsin. The culture medium was replaced every other day.

### Insulin secretion assay

Insulin secretion was determined in MIN6 cells, RINm5f cells and islets from SD rats. For GSIS assays, cells or islets were incubated for 1 h in KRB buffer (2.6 mM CaCl_2_·2H_2_O, 1.2 mM MgSO_4_·7H_2_O, 1.2 mM KH_2_PO_4_, 4.9 mM KCl, 98.5 mM NaCl and 25.9 mM NaHCO_3_ (all from Sigma, USA) supplemented with 20 mM HEPES (Sigma, USA) and 0.1% BSA (Sigma, USA)) at 37 °C, 5% CO_2_ and then incubated for 1 h in KRB buffer with 2.8 mM or 16.7 mM glucose^2^. The insulin concentration in the supernatants was determined using ultrasensitive mouse or rat insulin ELISA kits (Alpco, USA).

### Western blotting

Pancreas and β-cells were homogenized using a 26 G needle syringe in 10 mM HEPES, 1 mM MgCl_2_, 5 mM EDTA, 0.2% (v/v) Triton X-100 (Sigma, USA), 10% (v/v) glycerol (Sigma, USA), protease inhibitor cocktail (Sigma, USA), phosphatase inhibitor cocktails 1 and 2 (Sigma, USA), 250 mM PMSF (Sigma, USA), and 15 mM β-mercaptoethanol (Sigma, USA). Total protein content was determined with a BCA kit. Then, 30–50 μg of total protein was resolved on an 8–12% SDS-PAGE gel and transferred onto a PVDF membrane (Millipore, USA). Membranes were incubated in blocking buffer (20 mM Tris, 137 mM NaCl, 0.02% Tween 20, 5% BSA) for 2 h followed by incubation with the antibody anti-NMDAR1 (1:1000, Abcam, USA), IκB (1:1000, Proteintech, China), NLRP3 (1:1000, Abcam, USA) or cleaved caspase-3 (1:200, Abcam, USA). Band intensity was determined and quantified using an Odyssey IR scanner (Li-Cor Biosciences).

### Confocal microscope fluorescent images

Fluorescence intensity of intracellular [Ca^2+^] induced by fluo-3 AM in RINm5f cells was detected using a confocal microscope (Perkin Elmer UltraVIEW VoX, USA). Briefly, RINm5f cells were seeded onto glass coverslips in plates. After 24 h, the culture medium was removed. Cells were washed with PBS and incubated in HBSS with 5 μM fluo-3 AM (Sigma, USA) for 40 min at 37 °C. Fluo-3 AM solution was removed and cells were washed with HBSS or HBSS containing EGTA (4 mM).

### Cell viability assay

For the cell viability assay, an MTT (Sigma, USA) assay was performed. Cells were plated in 96-well plates (5 × 10^3^ per well) and incubated with medium containing various concentrations of NMDA (0, 0.1, 0.3, 1, 3 or 10 mM) for 72 h. Culture media was changed to serum-free media containing dissolved MTT (0.5 mg/mL). After 4 h, the media was removed and DMSO was added to each well. The optical density was measured at 492 nm using a microplate reader (Thermo, USA).

### LDH activity assay

Cells were plated in 96-well plates (5 × 10^3^ per well) and incubated with medium containing various concentrations of NMDA (0, 0.1, 0.3, 1, 3 or 10 mM) for 72 h. The cell medium was collected for measurement of LDH activity. LDH activity was determined using a Sigma Tox-7 *in vitro* toxicology kit and reported as the amount of LDH activity in the medium.

### Determination of cellular ATP level

For measurement of intracellular ATP, cells were incubated in KRB buffer for 1 h, followed by stimulation with glucose (16.7 mM) for 10 min. Cellular ATP levels were measured using a firefly luciferase-based ATP assay kit (Beyotime, China). The emitted light, which was linearly related to the ATP concentration, was measured using a multimode plate reader (Thermo Fisher Scientific, USA). The cellular ATP level was normalized to total protein determined by the BCA (Pierce, USA).

### Intraperitoneal glucose tolerance test (IPGTT) and insulin releasing test (IRT)

Mice were fasted for 12 h and then injected with glucose (2 g/kg) intraperitoneally. Glucose concentrations were measured in blood collected from the tail 0, 30, 60, 90 and 120 min after intraperitoneal injection. Glucose concentrations were measured twice at each time point using an automatic glucometer (Roche, Germany). Meanwhile, insulin concentrations were measured 15 min after intraperitoneal glucose injection with an ELISA (Alpco, USA).

### Lentivirus-mediated CRISPR/Cas9 knockdown of NMDAR1 expression

The CRISPR-Cas9 GluN1 sgRNA was purchased from Genechen (China). GluN1 sgRNA sequences were sgRNA1, CAAGATCGTCAACATCGGCG; sgRNA2, GTTGACGATCTTGGGGTCGC; sgRNA3, GTGGGAGTGAAGT GGTCGTT. RINm5f cells were infected with concentrated virus. The supernatant was replaced with complete culture medium after 24 h.

### Cell apoptosis

MIN6 cells were plated in 6-well plates (1 × 10^6^ per well) and incubated with glucose (33.3 mM) and/or MK801 (50 μM) for 72 h. Cells were collected and fluorescently labeled for detection of apoptosis by adding 500 μL of binding buffer, 5 μL of Annexin V-FITC and 5 μL of propidium iodide (Roche, USA) to each sample. Samples were mixed gently and incubated at room temperature in the dark for 15 min. Cells were immediately analyzed on flow cytometry (Beckman Coulter MoFloTM XDP, USA).

### Measurement of cytokine levels in serum by ELISA

Cells were plated in 6-well plates (1 × 10^6^ per well) and incubated with glucose (33.3 mM) and/or MK801 (50 μM) for 72 h. The supernatants of the cells were collected for the measurement of inflammatory cytokines (IL-1β and TNF-α) measurement using a commercially available ELISA kit (Sigma, USA), according to the manufacturer’s instructions.

### Glutamate content assay

Cells were plated in 6-well plates (1 × 10^6^ per well) and incubated with glucose (33.3 mM) for 72 h. The supernatants of the cells were collected for glutamate measurement using a glutamate detection kit (JianCheng, China). Mice were fasted for 12 h and then the serum was collect for glutamate measurement using a glutamate detection kit (JianCheng, China).

### Measurement of intracellular ROS

Intracellular ROS was determined by fluorometric assay (DCFH-DA assay, Sigma, USA). Cells were plated in 6-well plates (1 × 10^6^ per well) and incubated with glucose (33.3 mM) and/or MK801 (50 μM) for 72 h. Cells were loaded with 10 μM DCFH-DA for 20 min at 37 °C. The generation of ROS was determined using Varioskan Flash (Thermo Fisher Scientific, USA) with excitation and emission wavelengths at 488 and 525 nm, respectively. Relative ROS content was standardized to total protein content of the cells.

### Immunofluorescence

Pancreatic tissues were fixed overnight in 4% paraformaldehyde and then embedded in paraffin. Serial 4-μm sections were mounted on slides. Localization of insulin and cleaved caspase-3 in pancreatic islets was performed by a double-labeled immunofluorescence method. The slides were deparaffinized with sequential changes in xylene and rehydrated with descending concentrations of ethanol. This was followed by antigen retrieval, conducted by boiling the slides in citrate buffer followed by gradual cooling. After being washed in PBS, the sections were treated with a blocking agent (0.5% bovine serum albumin, Sigma, USA) for 45 min at room temperature. Sections were incubated overnight at 4 °C with a cocktail of two antibodies: rabbit anti-insulin antibody (1:50, Abcam, USA) and rat anti-cleaved caspase-3 antibody (1:20, Abcam, USA). Then, sections were incubated with FITC and Cy3-conjugated secondary antibody after washing in PBS. Sections were viewed with a fluorescence microscope (Thermo, USA). The intensity of cleaved caspase-3-positive signals in the insulin-positive area was measured using Image J software.

### Statistics

Results are shown as the means ± SEM. Statistical analysis was performed with SPSS17.0. Statistical evaluations were made by one-way ANOVA followed by Tukey’s method. *p* < *0.05* was considered statistically significant.

## Results

### Glutamate is increased in the plasma of diabetics *in vivo* and in the supernatant of RINm5f cells exposed to high glucose *in vitro*

**Figure 1:**
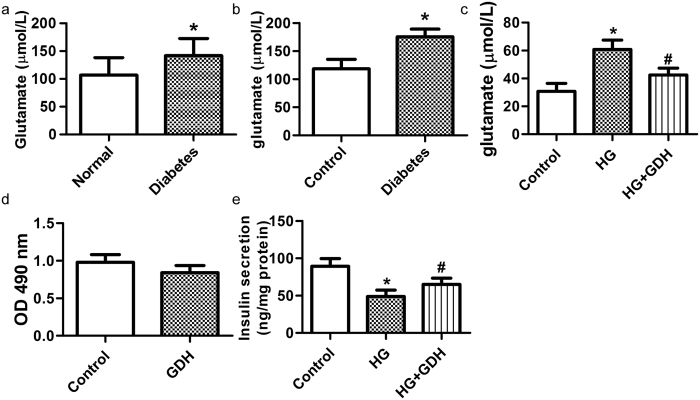
Glutamate was increased in the plasma of diabetics *in vivo* and in the supernatant of RINm5f cells exposed to high glucose *in vitro*. (**a**) The concentration of glutamate in the plasma of normal or diabetic patients (*p < *0.05* vs Normal). (**b**) The concentration of glutamate in the plasma of control or diabetic mice (n = 14, *p < *0.05* vs Control). (**c**) The concentration of glutamate in the supernatant of cultured RINm5f β-cells treated with 33.3 mM glucose for 72 h and/or GDH (20 unit/mL) (n = 5, *p < *0.05* vs control, ^#^*p* < *0.05* vs HG). (**d**) Effect of GDH (20 unit/mL) treatment for 72 h on the cell viability of RINm5f β-cells (n = 9). (**e**) Effect of GDH treatment on insulin secretion from RINm5f β-cells incubated with 33.3 mM glucose for 72 h (n = 5, *p < *0.05* vs control, ^#^*p* < *0.05* vs HG).

To investigate the change in glutamate in the plasma of diabetics, glutamate was measured. The plasma glutamate level was elevated in the diabetic patients and mice (Fig. 1a,b). In addition, high glucose is known to increase glutamate release in cerebral synaptosomes^21^,^22^ and β-cells share many features with neurons^23^. Our results indicated that the glutamate in the culture supernatant of RINm5f cells was increased under high-glucose conditions for 72 h (Fig. 1c). To further explore the effect of glutamate under high-glucose conditions on β-cell lines, L-glutamic dehydrogenase (GDH), which catalyzes the conversion of glutamate to α-ketoglutarate^24^, was applied to decompose the released glutamate in RINm5f cells. The result showed that GDH notably reduced the glutamate content in the supernatant of RINm5f cells under high-glucose conditions (Fig. 1c). GDH improved the GSIS from RINm5f cells under chronic high-glucose conditions (Fig. 1d,e). These data suggest that an increase of glutamate may contribute to RINm5f β-cell dysfunction and development of diabetes.

### Prolonged activation of NMDA receptors causes dysfunction in β-cell lines *in vitro*

**Figure 2:**
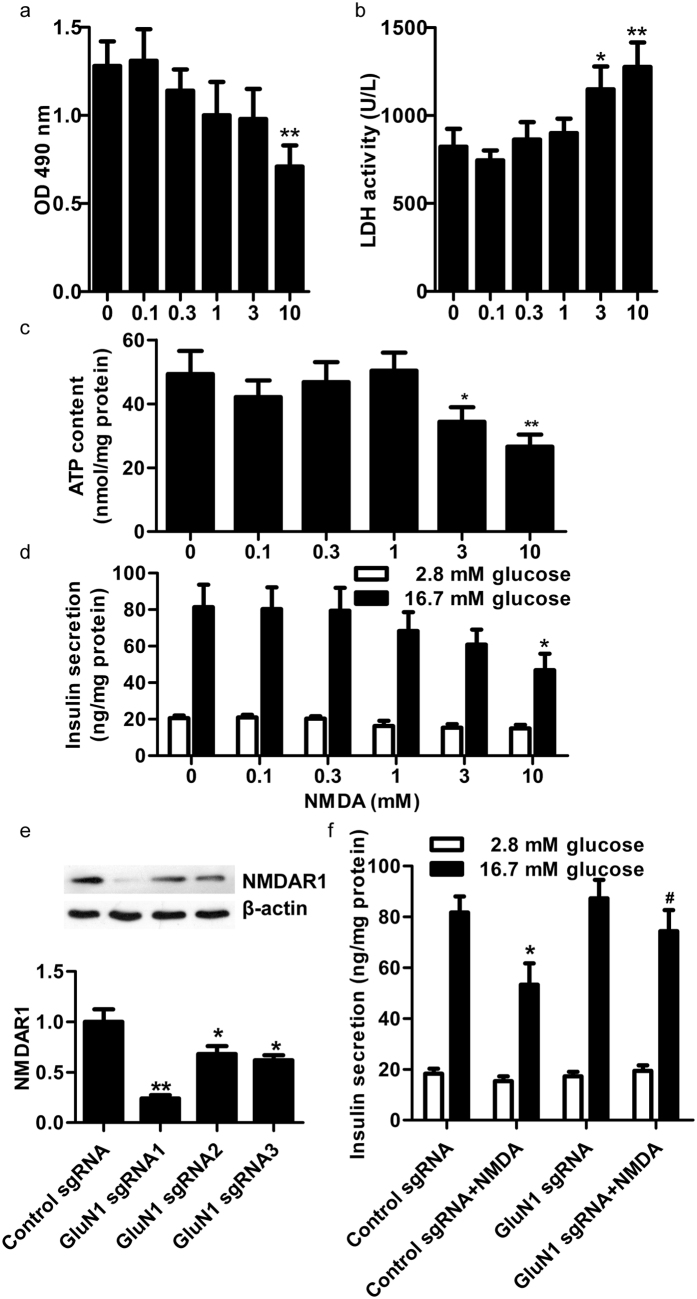
Prolonged activation of NMDA receptors causes β-cell dysfunction *in vitro*. (**a**) Effect of different concentrations of NMDA treatment for 72 h on the cell viability of RINm5f β-cells (n = 9, **p < *0.01* vs 0 mM NMDA group). (**b**) Effect of different concentrations of NMDA treatment for 72 h on the LDH activity in the supernatant of cultured RINm5f β-cells (n = 6, *p < *0.05* vs 0 mM NMDA group; **p < *0.01* vs 0 mM NMDA group). (**c**) Effect of different concentrations of NMDA treatment for 72 h on the intracellular ATP content of RINm5f β-cells (n = 5, *p < *0.05* vs 0 mM NMDA group; **p < *0.01* vs 0 mM NMDA group). (**d**) Effect of different concentrations of NMDA treatment for 72 h on insulin secretion from RINm5f β-cells (n = 6, *p < *0.05* vs 0 mM NMDA group). (**e**) Representative western blot showing the knockout efficiency of NMDAR1 protein in RINm5f cells treated with GluN1 sgRNA (n = 4, *p < *0.05* vs control sgRNA; **p < *0.01* vs control sgRNA group). (**f**) Insulin secretion from RINm5f cells transfected with sgRNA under stimulation with NMDA (10 mM) for 72 h (n = 6, *p < *0.05* vs control sgRNA, ^#^*p* < *0.05* vs control sgRNA + NMDA group).

It has been reported that short-term NMDA treatment increased transient secretion of insulin^10^. However, the effect of prolonged NMDAR activation in normal β-cells is still unclear. First, we confirmed the functional expression of NMDARs and mRNA expression of other glutamate receptors in the pancreas and β-cell lines of rat or mice (Supplemental Figures 1–3). We also detected the mRNA expression of glutamate receptors in islets of rats (Supplemental Figure 4). Then, we treated RINm5f cells with different concentrations of NMDA for 72 h. MTT assays revealed a dramatic reduction in the cell viability of RINm5f β-cells after 10 mM NMDA treatment (Fig. 2a). LDH activity in the supernatant of cultured RINm5f cells was significantly increased after exposure to NMDA (3 or 10 mM) (Fig. 2b). The synthesis of ATP in RINm5f cells was dramatically decreased after long-term pretreatment with NMDA (3 or 10 mM) (Fig. 2c). In addition, GSIS was markedly suppressed by long-term pretreatment with NMDA in a dose-dependent manner (1, 3 and 10 mM), while basal insulin secretion was not altered (Fig. 2d). These data indicate that long-term treatment with exogenous NMDA causes RINm5f β-cell dysfunction.

To test whether the NMDA-induced β-cells dysfunction was mediated by NMDARs, NMDAR1 protein was silenced using CRISPR-Cas9-sgRNA (Fig. 2e). We found that the GSIS of RINm5f cells treated with *GluN1*-knockout sgRNA was unaffected after exposure to NMDA for 72 h (Fig. 2f). Thus, the present data indicate that the dysfunction of islet β-cells induced by long-term exposure to NMDA is mediated by NMDAR.

### NMDAR inhibitor or *GluN1*-knockout sgRNA protects against glucotoxicity in islet β-cells

**Figure 3:**
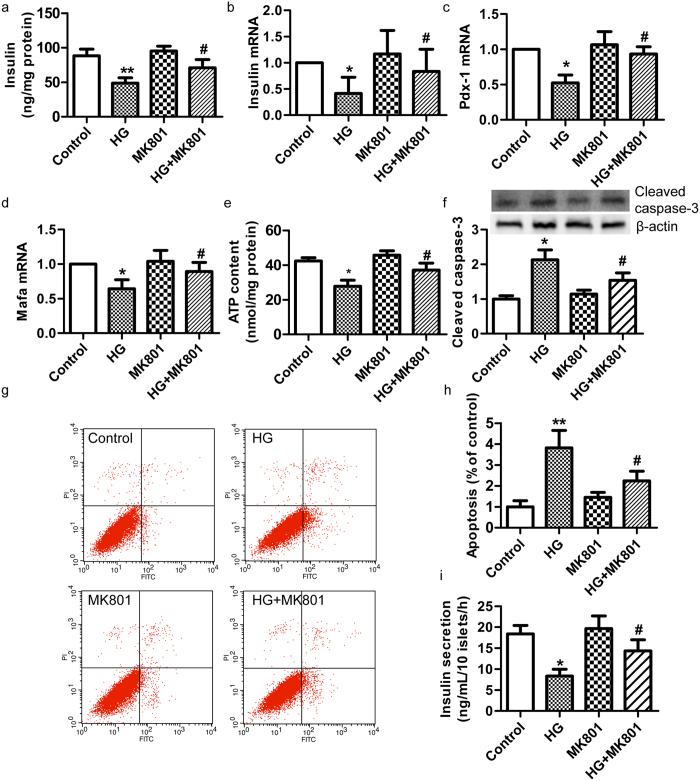
MK801 attenuated the high-glucose-induced dysfunction of MIN6 β-cells. After glucose (16.7 mM) stimulation for 1 h, MK801 (50 μM) increased the insulin secretion (**a**) and *Insulin* mRNA expression (**b**) in MIN6 β-cells exposed to high glucose for 72 h. (**c–d**) MK801 (50 μM) increased the mRNA expression of *Pdx-1* and *Mafa* in MIN6 β-cells exposed to high glucose for 72 h. (**e**) After glucose (16.7 mM) stimulation for 10 min, MK801 (50 μM) increased the intracellular ATP content of MIN6 β-cells exposed to high glucose for 72 h. (**f**) Representative western blot showing that MK801 reduced the cleaved caspase-3 protein in MIN6 β-cells exposed to high glucose for 72 h. (**g–h**) MK801 (50 μM) decreased the apoptosis of MIN6 β-cells exposed to high glucose for 72 h (n = 6, *p <* 0.05* vs control, **p <* 0.01* vs control, ^#^*p* <* 0.05* vs HG). **(i)** After glucose (16.7 mM) stimulation for 1 h, MK801 (50 μM) increased the insulin secretion in islets exposed to high glucose for 72 h (n = 4, *p <* 0.05* vs control; ^#^*p* <* 0.05* vs HG.).

As glucotoxicity plays a key role in the pathogenesis of diabetes^25^, we next tested whether endogenous glutamate contributes to the development of glucotoxicity through activation of NMDARs in β-cells. High-glucose culture (33.3 mM) for 72 h significantly decreased the GSIS from MIN6 cells, while a selective and specific antagonist of NMDAR^26^, MK-801 (50 μM), protected MIN6 cells against the impairment (Fig. 3a). The gene expression of *Insulin, Pdx*-1 and *Mafa*, involved in β-cell function, were significantly decreased by 33.3 mM glucose treatment for 72 h, while MK-801 abrogated this suppressing effect (Fig. 3b–d). Furthermore, we found that MK-801 dramatically attenuated the decrease of ATP content in MIN6 cells induced by high-glucose treatment for 72 h (Fig. 3e). The expression of cleaved caspase-3 protein and the rate of cell apoptosis were notably increased in MIN6 cells exposed to high glucose for 72 h. MK-801 also reduced the apoptosis and cleaved caspase-3 protein expression (Fig. 3f–h). Furthermore, we found that MK-801 dramatically attenuated the decrease in GSIS in islets induced by treatment with 33.3 mM glucose for 72 h (Fig. 3i). These data show that MK-801 mitigates the toxicity of chronic high glucose, enhances insulin secretion, and decreases the apoptosis of β-cells exposed to high glucose.

In contrast to MK-801, another NMDAR antagonist, Memantine, is a clinical drug that is used in humans in the treatment of Alzheimer’s disease symptoms^27^. Our results also showed that Memantine significantly improved the decline in GSIS from MIN6 cells induced by high-glucose treatment for 72 h (Supplemental Figure 5a,b). In addition, Memantine recovered *Insulin* gene expression (Supplemental Figure 5c) under high-glucose conditions for 72 h. In summary, Memantine, similar to MK-801, improves GSIS from β-cells under high-glucose conditions.

**Figure 4:**
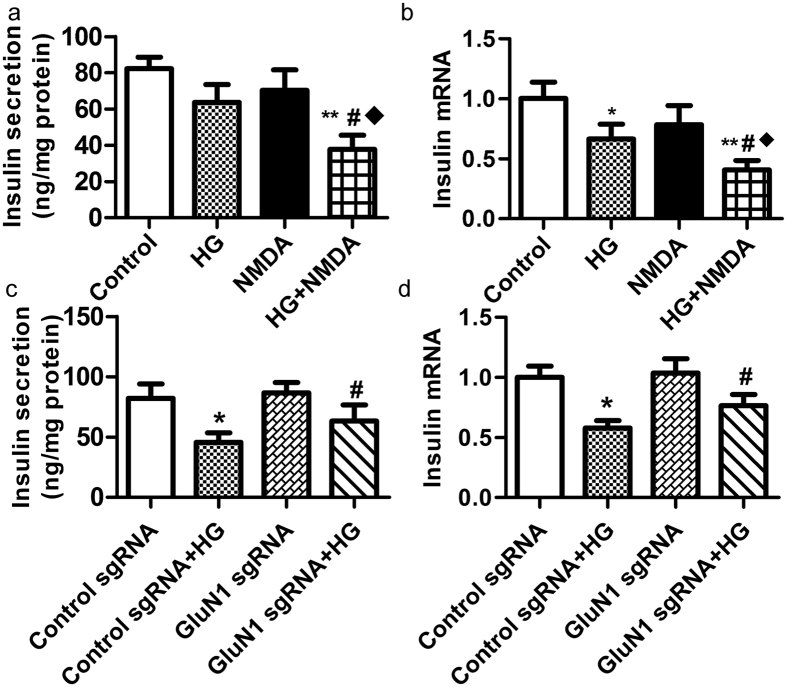
The effect of GluN1 sgRNA on RINm5f β-cells treated with 33.3 mM glucose for 72 h. (**a,b**) NMDA (3 mM) aggravated the decrease in insulin secretion and mRNA expression of *insulin* in RINm5f β-cells exposed to high glucose (33.3 mM) for 48 h (n = 5, *p <* 0.05* vs control, **p <* 0.01* vs control, ^#^*p* <* 0.05* vs HG, *p* <* 0.05* vs NMDA). (**c–d**) GluN1 sgRNA partially restored the insulin secretion from RINm5f cells exposed to 33.3 mM glucose (n = 5, *p <* 0.05* vs control sgRNA, ^#^*p* <* 0.05* vs control sgNRA + HG).

MIN6 β-cells were treated with 3 mM NMDA and 33.3 mM glucose for 48 h. The results showed that high glucose together with NMDA induced a more obvious reduction in both GSIS and *insulin* gene expression compared to β-cells treated with NMDA or glucose alone (Fig. 4a,b). To further determine whether the β-cell dysfunction induced by high glucose is mediated by NMDARs, NMDAR1 was silenced using sgRNA. RINm5f cells treated with knockout sgRNA showed a recovery in GSIS and *Insulin* gene expression in response to 33.3 mM glucose for 72 h (Fig. 4c,d). These observations suggest that high glucose-induced β-cells dysfunction is mediated, at least in part, by the increased release of endogenous glutamate through activation of NMDARs in an autocrine manner.

### NMDAR blockers decrease the expression of inflammatory cytokines and oxidative stress in high glucose-treated β-cells *in vitro*

**Figure 5:**
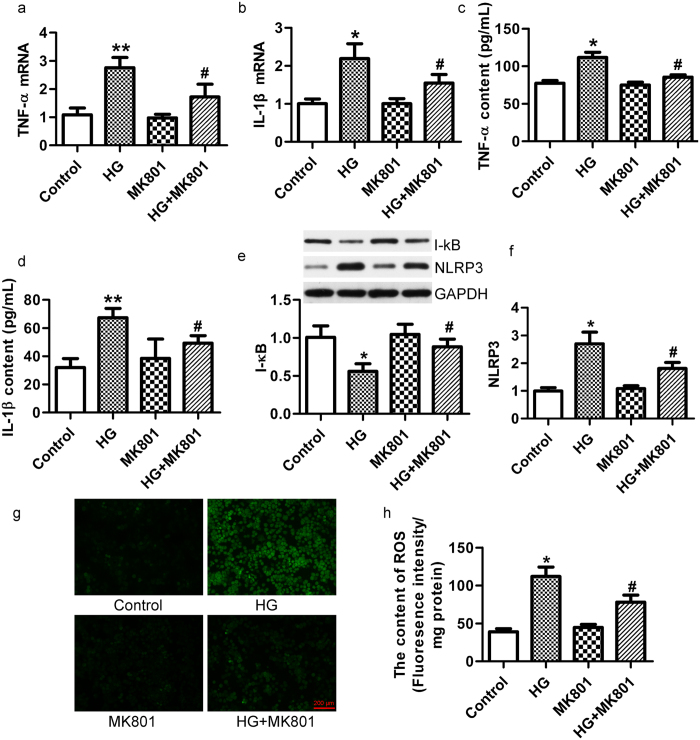
MK801 inhibited the expression of inflammatory cytokine and oxidative stress in high-glucose-treated β-cells. MK801 decreased the mRNA expression of *TNF-α* (**a**) and *IL-1β* (**b**) in MIN6 cells treated with glucose (33.3 mM) for 72 h, detected by real-time PCR. MK801 decreased the content of TNF-α (**c**) and IL-1β (**d**) protein content in the supernatant of MIN6 cells treated with glucose (33.3 mM) for 72 h, detected by ELISA. (n = 6, *p <* 0.05* vs control, **p <* 0.01* vs control, ^#^*p* <* 0.05* vs HG). (**e–f**) MK801 inhibited the degradation of I-κB and the NLRP3 expression in β-cells exposed to high glucose (33.3 mM) for 72 h. (**g,h**) MK801decreaed the production of ROS in RINm5f cells exposed to high glucose (33.3 mM) for 72 h (bar = 200 μm, n = 4, *p <* 0.05* vs control, ^#^*p* <* 0.05* vs HG).

It has been reported that inflammation contributes to the initiation, development and progression of diabetes^28^. We found that MK801 treatment reduced the mRNA and protein expression of TNF-α and IL-1β in MIN6 cells under high-glucose conditions *in vitro* (Fig. 5a–d). IL-1β stimulates inhibitor of κB (I-κB) degradation, which reflects the activation of the NF-κB pathway and increases expression of the NLRP3 inflammasome^29^. In this study, the degradation of I-κB and the expression of NLRP3 inflammasomes were significantly increased in MIN6 cells cultured with 33.3 mM glucose for 72 h. MK801 decreased I-κB protein degradation and NLRP3 protein expression (Fig. 5e,f). Oxidative stress aggravates β-cell apoptosis through the translocation of NF-κB^30^. We found that MK801 attenuated the production of ROS in RINm5f cells induced by 33.3 mM glucose for 72 h (Fig. 5g,h). These data indicate that NMDAR blockers can decrease the expression of inflammatory cytokines and oxidative stress in high glucose-treated β-cells.

### Memantine increases insulin secretion and the expression of genes associated with β-cell function in the pancreas of diabetic mice

**Figure 6:**
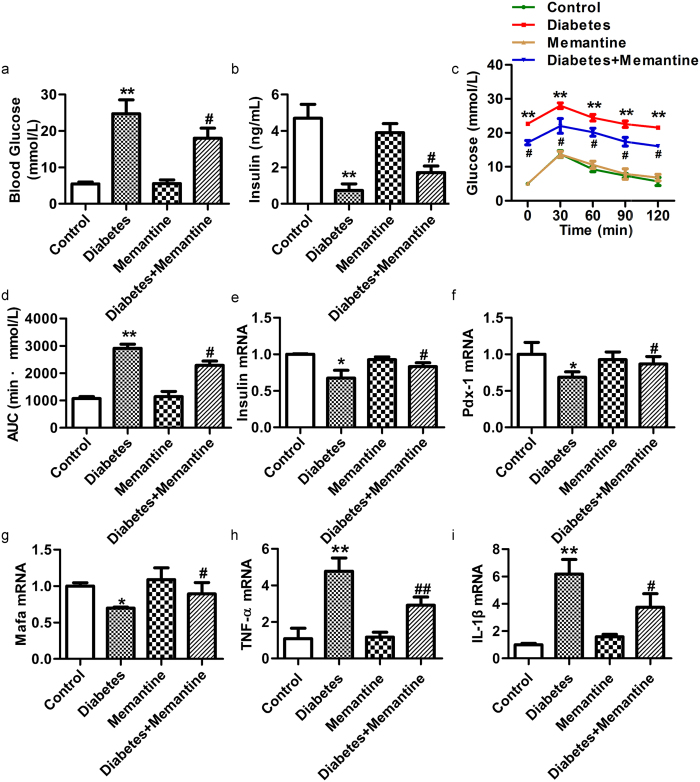
Memantine improved the function of pancreatic islets in diabetic mice. (**a**) Memantine treatment decreased the FBG concentration of diabetic mice. (**b**) Memantine increased the insulin content in serum of diabetic mice 15 min after intraperitoneal glucose injection. (**c**) Memantine improved glucose tolerance in diabetic mice. (**d**) Memantine treatment decreased the area under the curve of glucose tolerance after glucose injection. Memantine increased the mRNA expression of *Insulin* (**e**), *Pdx-1* (**f**) and *Mafa* (**g**) but decreased the mRNA expression of *TNF-α* (**h**) and *IL-1β* (**i**) in the pancreas of diabetic mice (n = 8–12, *p <* 0.05* vs control, **p <* 0.01* vs control, ^#^*p* <* 0.05* vs diabetes).

**Figure 7:**
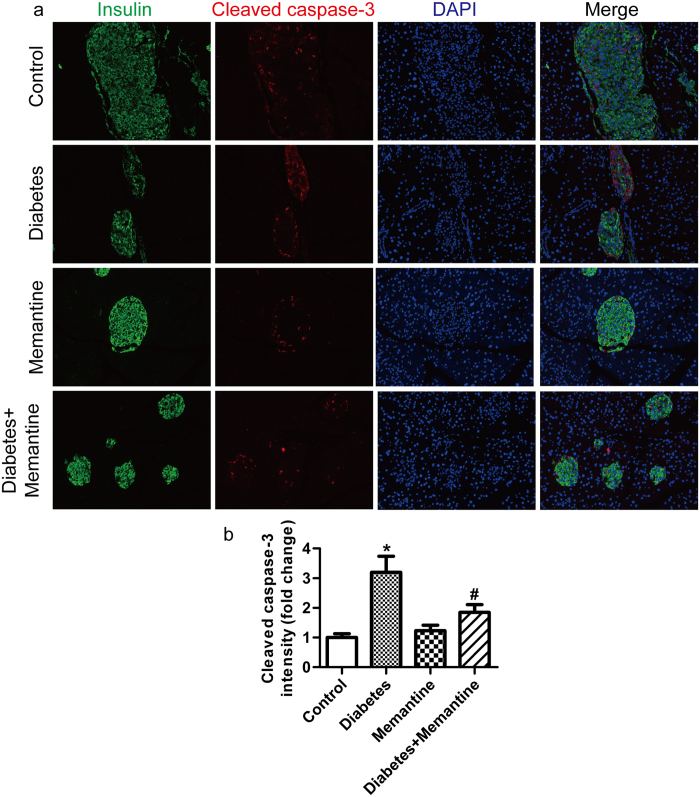
Memantine treatment inhibited the cleaved caspase-3 expression in rat islets. Immunofluorescence staining of pancreatic sections from control and diabetic mice treated with or without Memantine. Pancreatic sections were costained with anti-insulin (β cell; green) and anti- cleaved caspase-3 (red) antibodies (**a**), and the intensity of cleaved caspase-3-positive signals in the insulin-positive area was measured (**b**). (n = 5, *p <* 0.05* vs control, ^#^*p* <* 0.05* vs diabetes).

Further study was performed to examine the effect of NMDAR activation induced by endogenous glutamate on β-cell dysfunction in diabetic mice. Memantine (10 mg/kg, intraperitoneally injected daily for 21 days) significantly reduced FBG levels (Fig. 6a), but increased the insulin release in response to intraperitoneal injections of glucose (Fig. 6b) compared to diabetic mice without Memantine injection. Memantine also improved glucose tolerance in diabetic mice and significantly decreased blood glucose 30, 60, 90, 120 min after glucose injection (Fig. 6c). The area under the curve (AUC) for blood glucose after glucose injection in diabetic mice treated with Memantine was significantly decreased compared to that of untreated diabetic mice (Fig. 6d). Further more, the diabetic mice treated with Memantine expressed higher levels of *insulin, Pdx-1* and *Mafa* mRNA (Fig. 6e–g) in the pancreas, but lower levels of *TNF-α* and *IL-1β* mRNA (Fig. 6h,i). Immunofluorescence confirmed that the expression of cleaved caspase-3 protein was increased in islets of diabetic mice, and Memantine treatment significantly decreased the expression of cleaved caspase-3 protein in islets (Fig. 7a,b). Thus, these data indicate Memantine improves the function of diabetic mice islets *in vivo*.

## Discussion

In the present study, we confirmed a significantly higher plasma level of glutamate in diabetic patients and mice than that in non-diabetic controls, and glutamate was also increased in the supernatant of high-glucose-induced RINm5f β-cells *in vitro*. High-glucose-induced impairment of GSIS was attenuated by GDH treatment, which eliminates glutamate in the supernatant of β-cells. We further demonstrated that NMDARs were expressed in rodent pancreas and β-cells. Long-term treatment with NMDA reduced cell viability, ATP production in response to high glucose and GSIS in β-cells. The effects of NMDA were eliminated by sgRNA-targeted *GluN1* knockout in β-cells. Furthermore, NMDAR antagonists (MK-801 and Memantine) and *GluN1* knockout protected β-cells against both impaired GSIS and apoptosis induced by high glucose. Both antagonists also induced the recovery of gene expression related to β-cell function. Memantine increased GSIS and decreased inflammatory cytokines in diabetic mice. In addition, MK801 inhibited I-κB degradation and attenuated NLRP3 protein expression *in vitro*. Taken together, we found for the first time that the chronic high-glucose-induced release of glutamate and excessive activation of NMDARs caused dysfunction of pancreatic islet β-cells.

It has been reported that diabetes affects the metabolism of amino acids, including glutamate^5^,^6^. Systemic plasma glutamate levels are elevated in several diseases that are characterized by oxidative stress and inflammation^4^, such as obesity^31^. Our data showed that plasma glutamate levels were elevated in diabetic patients and diabetic mice. These results are consistent with previous studies, which demonstrated increased glutamate levels in the plasma or serum of diabetic subjects^4^,^6^.

Glutamate, a predominantly excitatory amino acid, is the primary neurotransmitter in the mammalian central nervous system^32^. There are controversial results regarding the impact of glutamate on insulin secretion. Under physiological conditions, glutamate, which functions as a modulator messenger between or within cells, plays an important role in regulating insulin secretion^7^,^33^. Our results demonstrated that the concentration of glutamate in the supernatant of β-cells culture media was increased after treatment with high glucose for 72 h. This finding suggests that glutamate is a signal transmitter in islets through autocrine or paracrine way^7^. It has been reported that kainite and AMPA stimulate insulin secretion from isolated islets but NMDA does not affect insulin secretion^14^. Another study has shown that brief treatment with NMDA in the presence of 3.3 mM glucose also stimulates insulin secretion from MIN6 β-cells^10^. These results suggested that brief exposure to glutamate stimulated insulin secretion from β-cells. However, Boonnate has reported that daily consumption of dietary glutamate decreased the pancreatic β-cell mass in adult rats^34^. The present data showed that chronic high-glucose treatment doubled the glutamate content in culture media of RINm5f cells, indicating that endogenous glutamate may be released from β-cells under chronic hyperglycemic conditions. GDH, which decomposes the released glutamate, improved the GSIS of β-cells under high-glucose conditions. These findings indicate that the excessive release of endogenous glutamate plays an important role in the dysfunction of β-cells induced by prolonged high-glucose exposure.

Glutamate acts through specific glutamate receptors. Recently, functional glutamate receptors have been demonstrated in non-neuronal tissues and cells, including the kidney and lung, as well as in pancreatic β-cells^10^,^11^,^12^,^14^. mRNAs representing almost all of the glutamate receptors were expressed in the rat/mice pancreas/islet and RINm5f/MIN6 cells. Major glutamate neurotoxicity is mediated by the NMDAR^9^. We found that brief exogenous NMDA exposure caused a transient rise of [Ca^2+^]i in RINm5f cells. In addition, we also showed that mRNAs of glutamate receptors were also expressed in islets of rats. These findings suggest functional expression of multiple types of glutamate receptors in pancreatic β-cells.

Brief treatment with NMDA can increase insulin secretion^10^, while a recent study showed that a NMDAR inhibitor (DXM) increases insulin secretion, showing a beneficial effect of NMDAR inhibitors on islets in type 2 diabetic mice and patients^16^. This suggests that increases in plasma glutamate in diabetes play an important role in the dysfunction of β-cells through activation of NMDARs. However, the effect of prolonged NMDAR activation in normal islet β-cells is still unclear. In our *in vitro* studies, we demonstrated that treatment with exogenous NMDA for 72 h reduced cell viability, the production of ATP and GSIS of RINm5f β-cells. In contrast, *GluN1* knockout eliminated these adverse effects of exogenous NMDA on RINm5f cells. These findings indicate for the first time that long-term treatment with exogenous NMDA causes dysfunction of RINm5f β-cells through the activation of NMDAR, similar to neuro-excitotoxicity in neurons.

Glucose is the most important physiological factor involved in the regulation of insulin secretion^35^. It is well established that physiological regulation of glucose is maintained by β-cell function. In contrast, prolonged or repeated exposure to elevated glucose concentrations exerts deleterious effects on β-cell function, which is called glucotoxicity^36^. Previous research has suggested that β-cells incubated with high glucose for 72 h exhibited impaired GSIS and survival^37^. We found that NMDAR blockers and *GluN1* knockout protected β-cells against the impairment in GSIS and the apoptosis induced by chronic high glucose, and thus attenuated the glucotoxicity of β-cells. These results showed for the first time that NMDAR activated by release of endogenous glutamate in an autocrine/paracrine manner mediated β-cell dysfunction induced by chronic high glucose.

In the CNS, the excessive activation of NMDARs leads to oxidative damage^38^, occurring along with increased ROS production and causing excitatory neurotoxicity in neurons^39^. Several lines of evidence indicate that NMDARs also play an important role in regulating inflammation in neuronal and non-neuronal tissues, such as pulmonary inflammation^17^,^18^. In the present study, the results showed that inhibition of NMDARs significantly reduced the chronic high-glucose-induced increase in the expression of TNF-α and IL-1β in MIN6 cells. Elevated glucose concentrations increased the formation of ROS and the production of mature IL-1β, thereby resulting in β-cells dysfunction^40^,^41^,^42^. In addition, excessive activation of NMDARs increases the production of ROS in neural cells^43^. Our data demonstrated that MK801 attenuated the increase of ROS induced by prolonged high glucose in β-cells. NMDAR-mediated activation of NF-κB has been reported in the rat brain following ischemia^44^. In our *in vitro* studies, glucotoxicity induced the degradation of I-κB and the activation of NLRP3 inflammasomes, which were suppressed by MK-801. These data suggested that glutamate released from β-cells under high-glucose conditions increased the production of ROS and promoted the activation of NF-κB and NLRP3 inflammasomes, accelerating the dysfunction of β-cells through excessive activation of NMDA receptors.

We have demonstrated that increased release of glutamate resulted in glucotoxicity in β-cells through excessive activation of NMDAR *in vivo*. We next investigated the role of Memantine in the treatment of diabetic mice. Our study showed that treating diabetic mice with Memantine could mitigate the impairment of GSIS and attenuate the decrease in the expression of genes involved in β-cell function (*Insulin, Pdx-1* and *Mafa*). These findings were consistent with the study of Marquard, which showed that DXM increased insulin secretion in diabetic mice and patients^16^. In addition, our date suggested that Memantine reduced *TNF-α* and *IL-1β* mRNA levels in the pancreas of diabetic mice. These data indicated that increase in endogenous glutamate levels aggravated the β-cell dysfunction induced by glucotoxicity *via* excessive activation of the NMDARs *in vivo*. In diabetes, the inability of β-cells to secrete adequate amounts of insulin leads to the development of hyperglycemia. Hyperglycemia promotes the release of glutamate through autocrine or paracrine signaling. Excessive activation of NMDARs by increased glutamate accelerates the damage to β-cells, creating a vicious cycle. Blockade of NMDARs may interrupt this circle, and thus, may be a potential drug target for the treatment of diabetes.

In summary, this report demonstrates for the first time that long-term stimulation of β-cell lines with NMDA causes β-cell dysfunction through excessive activation of NMDARs. In the development of glucotoxicity in diabetes, endogenous glutamate aggravates the high-glucose-induced β-cells dysfunction *via* excessive activation of NMDA receptors on β-cells, leading to activation of NF-κB and NLRP3 inflammasomes. NMDA receptor blockade or knockout significantly improves β-cells function under high-glucose conditions *in vitro* and hyperglycemia *in vivo*. NMDARs may serve as a potential drug target in the treatment of diabetes.

## Additional Information

**How to cite this article:** Huang, X.-T. *et al*. An excessive increase in glutamate contributes to glucose-toxicity in b-cells via activation of pancreatic NMDA receptors in rodent diabetes. *Sci. Rep.* **7**, 44120; doi: 10.1038/srep44120 (2017).

**Publisher's note:** Springer Nature remains neutral with regard to jurisdictional claims in published maps and institutional affiliations.

## Supplementary Material

Supplementary Information

## Figures and Tables

**Table 1:** Characteristics of normal individuals and patients in the study.

	Normal (n = 20)	Diabetes (n = 32)
Age	64.31 ± 5.99	64.97 ± 6.67
Male	60% (12/20)	62.5% (20/32)
BMI	24.64 ± 2.38	24.46 ± 3.01
HbA_1C_	5.54 ± 0.23	6.57 ± 0.52
