# Supplementary material for: An excessive increase in glutamate contributes to glucose-toxicity in β-cells via activation of pancreatic NMDA receptors in rodent diabetes
Source: Sci Rep. 2017 Mar 17;7:44120. doi: 10.1038/srep44120 (PMC5356012; doi:10.1038/srep44120)
Supplement: Supplementary Information [file srep44120-s1.pdf]

An excessive increase in glutamate contributes to glucose-toxicity in β-cells via activation of pancreatic NMDA receptors in rodent diabetes

Xiao-Ting Huang[1], Chen Li[1,3], Xiang-Ping Peng[1], Jia Guo[1,5], Shao-Jie Yue[2], Wei Liu[1,5], Fei-Yan Zhao[1], Jian-Zhong Han[1], Yan-Hong Huang[1], Yang-Li[1], Qiang-Mei Cheng[1], Zhi-Guang Zhou[6], Chen Chen[4], Dan Dan Feng[1*], Zi-Qiang Luo[1*]

1 Department of Physiology, Xiangya School of Medicine, Central South University, Changsha, Hunan, China;

2 Department of Pediatrics, Xiangya Hospital, Central South University, Changsha, Hunan, China;

3 Department of Physiology, Changzhi medical college, Changzhi, Shanxi, China;

4 SBMS, The University of Queensland, Brisbane, Australia;

5 Xiangya Nursing School, Central South University, Changsha, Hunan, China;

6 Department of Metabolism and Endocrinology, the Second Xiangya Hospital, Central South University, Changsha, Hunan, China

* To whom correspondence should be addressed:

Prof. Zi-Qiang Luo or Associate Prof. Dan Dan Feng

Department of Physiology

Central South University Xiangya Medical School

Changsha, Hunan 410078, China

Tel.: +86-731-82355051; Fax: +86-731-82355056

E-mail: luozq1962@163.com or fengdandanph@163.com

Supplementary Figure 1

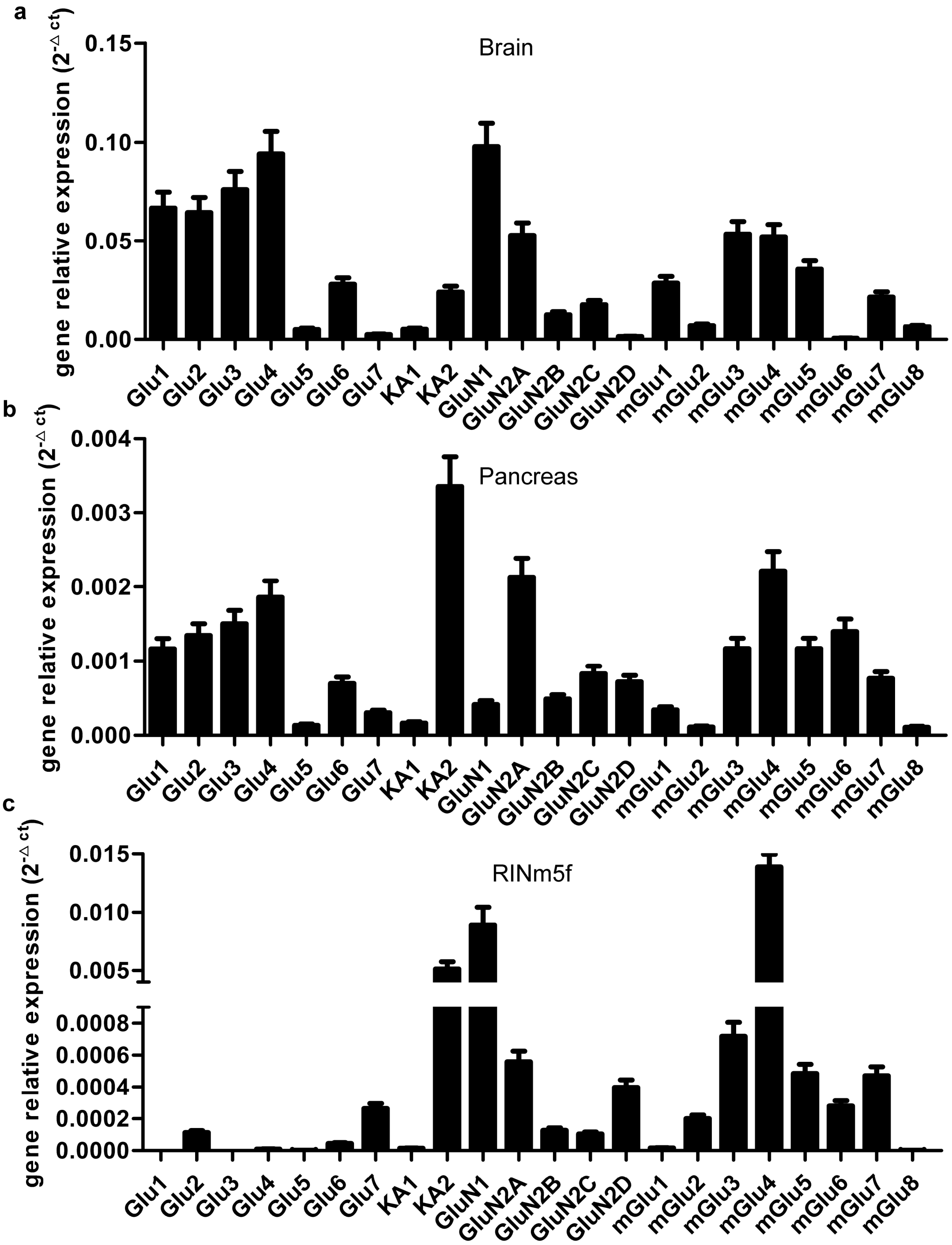

Real-time PCR analysis of the glutamate receptors mRNA expression in brain of rat (a), pancreas of rat (b) and pancreatic islet β-cell line RINm5f cell (c). Values are the means±SEM from each group (n=10).

Supplementary Figure 2

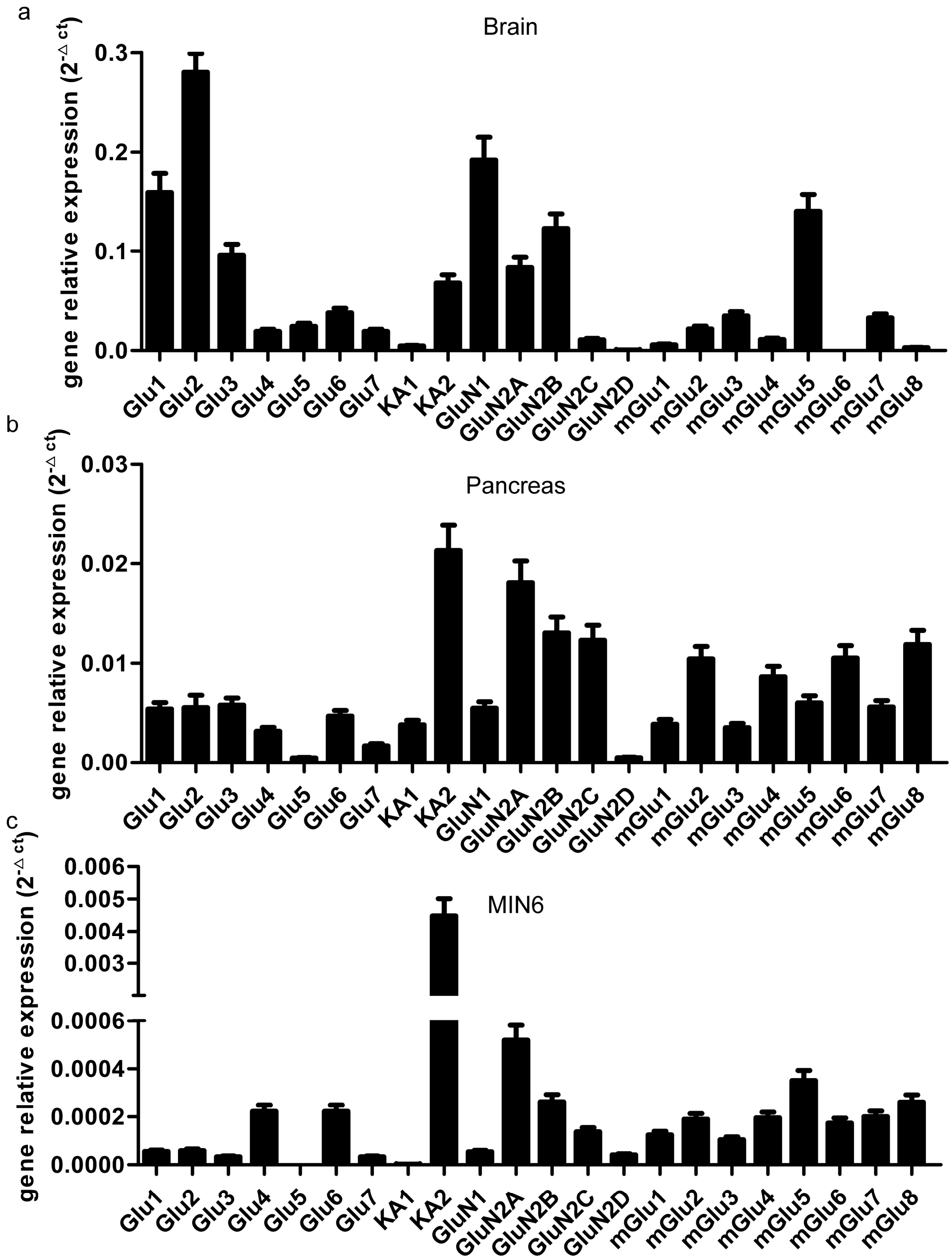

Real-time PCR analysis of the glutamate receptors mRNA expression in brain of mouse (a), pancreas (b) and pancreatic islet β-cell line MIN6 cell (c). Values are the means±SEM from each group (n=10).

Supplementary Figure 3

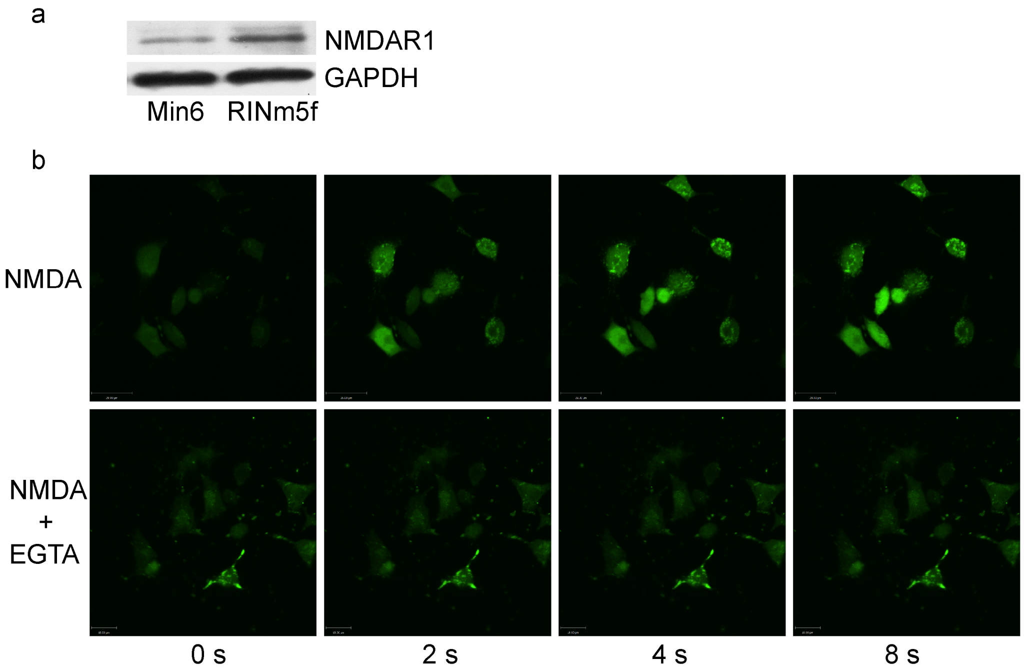

NMDA increases the calcium ion in RINm5f cells. The content of NMDAR1 protein in MIN6 and RINm5f cells (a). Intracellular calcium level in RINm5f cells in the presence of NMDA (3 mM) without or with EGTA (4 mM) (b).

Supplementary Figure 4

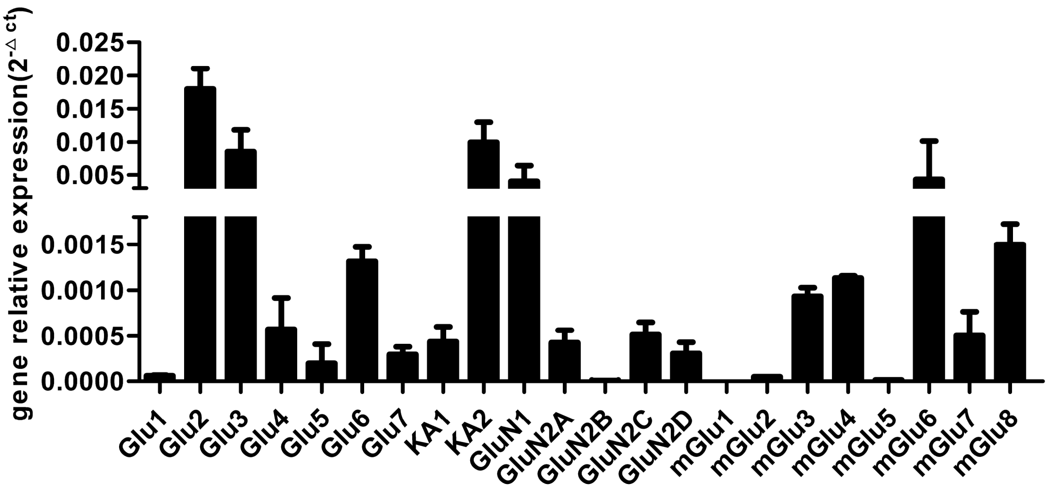

Real-time PCR analysis of the glutamate receptors mRNA expression in islets of rats. Values are the means±SEM from each group (n=4).

Supplementary Figure 5

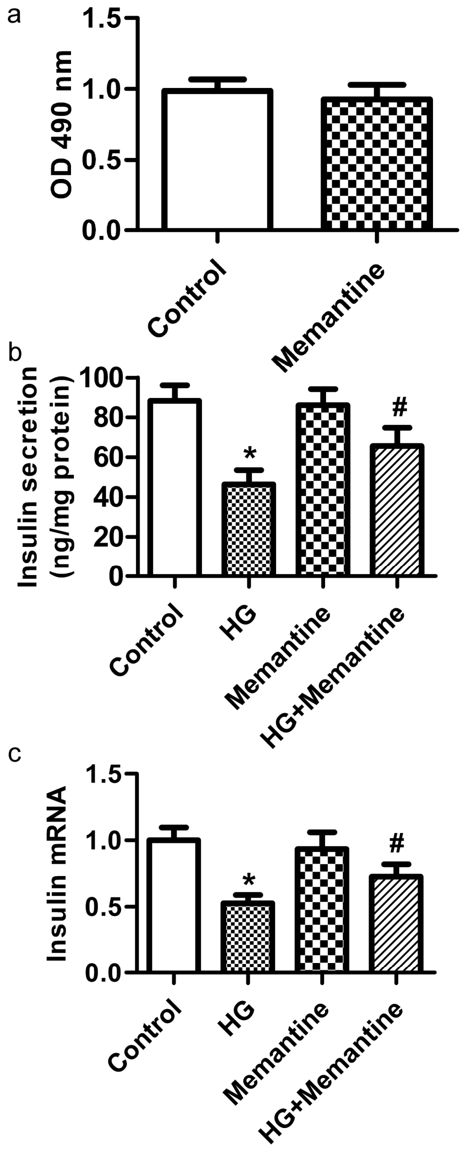

Memantine enhances insulin secretion and improves genes expression of MIN6 cells treated with 33.3 mM glucose for 72 h. MTT of MIN6 β-cells treated with memantine 50 μM for 72 h (a). Insulin secretion from MIN6 β-cells incubated in high glucose (33.3 mM) without or with memantine (b). The mRNA level of *Insulin* (c) gene in MIN6 cells treated as described in (b). (n=5.*P<0.05 vs control. #P<0.05 vs HG.)

Supplementary Table1. Sequence of specific primers used in this study

Gene	Species	Forward primer's sequence (5' to 3')	Reverse primer's sequence (5' to 3')	Length (bp)
GLUR1	mouse	GCTAGAAAAGAACGGCATCG	TCACTTGTCCTCCACTGCTG	168
GLUR2	mouse	AAAGGAGGAAAGGGAAACGA	GAACCATCCCTACCCGAAAT	203
GLUR3	mouse	CTCCTGATCCTCCCAATGAA	TCAGGAAAGCAGCAAGGTTT	183
GLUR4	mouse	AAGGCCGTACACACAGGAAC	CCTTCCTGCTCAGTCAAAGG	193
GLUR5	mouse	CACATTCAGACTCGCTGGAA	CTGGAGGGAGCTTTGATGAG	197
GLUR6	mouse	CCACTATTCCACGCATCCTT	GATCCACAACAAGCAGAGCA	170
GLUR7	mouse	ATGACAAAGGCCAGTGGAAC	AGAAGACACTGGGGTTGGTG	183
KA1	mouse	AATGGGTTTCAGCAGATTGG	GTGGTTCCCCTTCAGCATTA	156
KA2	mouse	AGTACGAGACCACGGACACC	CGAAGCGAAGGTACTGAAGG	177
GluN1	mouse	ACTCCCAACGACCACTTCAC	GTAGACGCGCATCATCTCAA	186
GluN2A	mouse	AGACCTTAGCAGGCCCTCTC	CTCTTGCTGTCCTCCAGACC	153
GluN2B	mouse	CCGCAGCACTATTGAGAACA	ATCCATGTGTAGCCGTAGCC	213
GluN2C	mouse	ATCGGGGTCAACAATACCAA	CACAGCAGAACCTCCACTGA	186
GluN2D	mouse	TAGTGTCAGTGCGCAGATCC	TCCTGGCAGAAGAAGTGGTT	178
mGLUR1	mouse	CCCTTTACAACGTGGAGGAA	GAACAAGGGCGTCTCTTCTG	152
mGLUR2	mouse	CTAGAGGCCATGCTTTTTGC	TAGCTGTGGGAGCATCACTG	223
mGLUR3	mouse	GAAAGGACAAAGGCAGCAAG	GCTAAAAGAGCCCGTCACTG	163
mGLUR4	mouse	GATATCAGGAGGGTGCTGGA	TCCTCTTGGGAAGAATGGTG	151
mGLUR5	mouse	AGACCAACCGTATTGCAAGG	AGACTTCTCGGATGCTTGGA	195
mGLUR6	mouse	GTGGGAGTGATAGCGTGGTT	TGACCATGAGCAGGAGACTG	163
mGLUR7	mouse	CCTGTCTTCCTGGCAATGTT	GGCTTGGCAATCATTAGGAA	170
mGLUR8	mouse	CATGTACACCACGTGCATCA	TGAACGTTCTGCTCTGGATG	192
β-actin	mouse	TTCCAGCCTTCCTTCTTG	GGAGCCAGAGCAGTAATC	182
GLUR1	rat	GGATACCGGATGCTCTTTCA	CCCGATGCCATTCTTTTCTA	126
GLUR2	rat	CTACGACGATTCCCTGGTGT	CTCTGCTTCCGAAGGTTACG	162
GLUR3	rat	TCAGGAAAGCAGCAAGGTTT	CTCCTGATCCTCCCAATGAA	183
GLUR4	rat	TTTGCAGGCAGATTGTCTTG	GGGGCTGGTGTTATGAAGAA	155
GLUR5	rat	TCCATCAGCAGTGCGTAGTC	ATCTGGCCAAACAAACCAAG	196

GLUR6	rat	GCTCATAAACGCCCACATTT	GCTAACCTAGCCGCCTTTCT	174
GLUR7	rat	CCCACACCTCTTTTCGTGTT	CCCATGAGAGCAGAGACTCC	195
KA1	rat	CCCTCCTCTGTGCTCTTCAC	GCTCCAGCATGACCTTCTTC	109
KA2	rat	AGGGGGTTGTGTCTGTCTTG	GCAAAGCGGAGGTACTGAAG	139
GluN1	rat	CGGCTCTTGGAAGATACAGC	GTGGGAGTGAAGTGGTCGTT	156
GluN2A	rat	ACATTGCAGAAGCTGCCTTT	TTCTGTGACCAGTCCTGCTG	186
GluN2B	rat	GTGAGAGCTCCTTTGCCAAC	TGAAGCAAGCACTGGTCATC	157
GluN2C	rat	AGACCAATACCCACCCTTCC	GTTGAGCACAGCAGCATCAT	187
GluN2D	rat	TAGTGTCAGTGCGCAGATCC	ACCATGAACCAGACGTAGCC	114
mGLUR1	rat	AACAAAAGCGGAATGGTACG	ACAGGCTCTGCAGGTGAACT	153
mGLUR2	rat	TTCATCGGCTTCACCATGTA	TGGAAAAGGATGATGTGCAA	179
mGLUR3	rat	TGCTGACCAAGACAAACTGC	AGGATAAGCCACACGGACAC	151
mGLUR4	rat	CAGGTTATGCCACACACAGG	GCAAAGACGGAAGAGAAACG	178
mGLUR5	rat	AGACCAACCGTATTGCAAGG	AGACTTCTCGGATGCTTGGA	195
mGLUR6	rat	AATGGACAGATGCTCCAAGG	AAGTGAGGAAGAGCGGAACA	120
mGLUR7	rat	AACCTGCTGCCCAATGTAAC	CGGAGGTGTCCTTCTGGATA	118
mGLUR8	rat	CCCTGGGATGGAATTATGTG	TCACGTGGGATTTTCTGTGA	133
β-actin	rat	GTCGTACCACTGGCATTGTG	CTCTCAGCTGTGGTGGTGAA	181
Insulin	mouse	GTTCACATGTGCAGGAATGG	CTGTGCTTTGGATGGGTTTT	170
Insulin	rat	TAGGACAGGGTCCCAGACAC	GCTTCTGCCAAGACCTTCAC	159
Pdx1	mouse	ACACAGCTCTACAAGGACCC	ACTTCCCTGCTCCAGTGATC	153
Mafa	mouse	AAATACGAGAAGTTGGCGGG	CACAGAAAGAAGTCGGGTGC	125
TNF-α	mouse	TCTCATTCCTGCTCGTGG	CTCTGCTTGGTGGTTTGC	200
IL-1β	mouse	GCCCATCCTCTGTGACTCAT	AGGCCACAGGTATTTTGTCG	230
